# IRW and IQW Reduce Colitis-Associated Cancer Risk by Alleviating DSS-Induced Colonic Inflammation

**DOI:** 10.1155/2019/6429845

**Published:** 2019-10-24

**Authors:** Yong Ma, Hongmei Jiang, Jun Fang, Gang Liu

**Affiliations:** ^1^College of Bioscience and Biotechnology, College of Animal Science and Technology, Hunan Agricultural University, Changsha, Hunan 410128, China; ^2^Key Laboratory of Animal Nutritional Physiology and Metabolic Process, Key Laboratory of Agro-Ecological Processes in Subtropical Region, Institute of Subtropical Agriculture, Chinese Academy of Sciences, National Engineering Laboratory for Pollution Control and Waste Utilization in Livestock and Poultry Production, Changsha, Hunan 410125, China

## Abstract

**Background and Objective:**

Bioactive peptides exert great influence in animals and human health by targeting gastrointestinal tracts. The colitis model of mice was induced by dextran sulfate sodium (DSS). Thirty-two 8-week-old mice weighing 23 g on average were randomly assigned to four groups of 8 each: mice fed basal diet (CON), mice fed basal diet with 5% DSS (DSS), mice fed 0.03% IRW with 5% DSS (IRW-DSS), and mice fed 0.03% IRW with 5% DSS (IQW-DSS). After an adaptation period of 3 days, on day 8, all mice were slaughtered. Serum samples were collected to determine the level of amino acids; colonic tissue was quick-frozen for the determination of gene expression.

**Methods:**

The aim of this study was to assess the ability of two kinds of peptides (IRW and IQW) to repair intestinal inflammatory in the DSS-induced model in accordance with serum amino acids and intestinal inflammatory factors.

**Results:**

The results demonstrated that the addition of IRW and IQW had a mitigating effect on DSS-induced intestinal inflammation. The level of Asp decreased in the serum of mice supplemented with IRW-DSS (*P* < 0.05), and IQW enhanced the level of Leu, but lowered the level of Ser (*P* < 0.05). IQW and IRW addition reduced the level of TNF-*α* and IL-17 (*P* < 0.05). No other significant effects were observed.

**Conclusions:**

The present study demonstrated that intracolic administration of IRW and IQW might be a novel option for preventing inflammatory bowel disease via regulating the level of serum amino acid and enhancing the intestinal immune defense.

## 1. Introduction

The main symptoms of inflammatory bowel disease (IBD) include Crohn's disease (CD) and ulcerative colitis (UC), which influence the gastrointestinal tract and cause recurrent diarrhea. The process of IBD is precipitated by a complex interaction of environmental, genetic, and immunoregulatory factors. The pathogenesis of IBD remains unclear, and the activation of inflammation and the recurrence of certain diseases are associated with natural and acquired immune responses, such as the overproduction of TNF-*α* and IFN-g in the intestine. Clinical data show that more than a quarter of cancers are caused by inflammation and chronic infections [1]. Accumulating experimental results confirm such associations; inflammation plays an important role in the cancer development process. Moreover, nearly 20% of these people with IBD are eventually suffering from CAC (colitis-associated cancer), showing a strong correlation between colonic inflammation and CAC risk. This is because activated proinflammatory cytokines can induce DNA damage and genomic mutations while promoting intestinal mucosal epithelial cell apoptosis and disrupting its signaling pathway [2]. Therefore, proinflammatory cytokines are important inducers in the development of CAC.

In animal experiments, inflammation model induced by DSS indicates altered intestinal permeability and reduced tight junction protein leads to impaired intestinal barrier function and commensal bacteria translocation to underlying lamina propria [3]. This process ultimately increases the expression levels of endotoxin and proinflammatory cytokines in the intestine [4]. The occurrence of CAC is inextricably linked to circulating proinflammatory cytokines. Serum metabolic profiles of human or animal models are usually used to investigate inflammatory diseases, such as IBD [5], rheumatoid arthritis [6], and chronic lymphocytic leukemia [7]. A multivariate index consisting of plasma amino acids can be used to diagnose and detect IBD, which provides us with new insights into the pathophysiology of the disease [5, 8]. Amino acid metabolism is disturbed in patients with IBD, and amino acid profiles may reflect the individual nutritional conditions and the activity of the disease [9–11]. The results of serum amino acids in ICR (Institute for Cancer Research) mouse experiments show that DSS (dextran sulfate sodium salt) treatment significantly alters the levels of amino acids in the serum, such as Trp (tryptophan), Glu (glutamic acid), and Gln (glutamine), which can be related to related signaling pathways in the colon [11, 12].

Mouse models of CAC and IBD have been commonly used to screen for the treatment of intestinal inflammation-related diseases. Egg protein transferrin derivative peptides (IQW and IRW) exhibit satisfactory efficacy in the treatment of TNF-induced inflammation, reflecting their health potential as food additives [13, 14]. Accumulated evidence has shown that bioactive peptides can regulate and alleviate intestinal inflammation. Through mouse simulation experiments, it was found that porcine *β*-defensin 2 can alleviate intestinal mucosal damage and regulate intestinal permeability through NF-*κ*B (nuclear factor kappa-light-chain-enhancer of activated B cells) signaling pathway to achieve the effect of alleviating IBD [15]. Our recent study found that two protein peptides, IRW and IQW, can alleviate colonic inflammation induced by *Citrobacter rodentium* [16]. However, there are few studies on serum amino acid changes in the DSS-induced inflammation model. In this study, we analyzed the effects of two peptides (IRW and IQW) on the amino acid level of serum and the expression of related inflammatory factors and barrier functional genes in colon tissues in the DSS-induced model.

## 2. Materials and Methods

### 2.1. Animal and Experimental Design

IRW and IQW are synthesized by Ontores (Zhejiang Province, China). The purity of the two small peptides is 99%. Thirty-two 8-week-old mice weighing on average 23 g were randomly assigned to four groups of 8 each: basal diet [17, 18] (CON), basal diet with 5% DSS (Meilun Biotechnology, Dalian Province, China, M.W: 5000), 0.03% IRW with 5% DSS (IRW-DSS), and 0.03% IRW with 5% DSS (IQW-DSS). The animals were housed with a 12-h light/dark rotation (the lighting time is 8 a.m.); the ambient temperature was controlled at 22–24°C and the humidity 55%–60%. After an adaptation period of 3 days, 5% DSS was diluted in water and all mice were fed the basal diet and drinking their respective water ad libitum; daily weight changes of mice were recorded. DSS was used for 7 days in mice, and on day 8, all mice were slaughtered. Serum samples were collected to determine the level of amino acids; colonic tissue was quick-frozen for the determination of gene expression. This study was carried out by the Chinese Guidelines for Animal Welfare and approved by the Animal Care and Use Committee of the Institute of Subtropical Agriculture, Chinese Academy of Sciences (0207).

### 2.2. Serum Amino Acid Analysis

The blood sample was collected at the same time point in each group before slaughter; then, the samples were centrifuged at 3000 rpm for 15 minutes at 4°C, and the upper serum was collected and stored at −20°C until use. In a sealed centrifuge container, 2.7 ml of sulfosalicylic acid was added to 300 *μ*l serum. The mixed solution was centrifuged at 1000 rpm for 10 minutes at 4°C after shaking and kept at rest for 15 min. The supernatant was percolated by 0.45 *μ*m membrane and measured by Hitachi L-8900 automatic amino acid analyzer.

### 2.3. Real-Time Quantitative

According to the manufacturer's instructions, the total RNA was extracted from the frozen colonic tissue by using TRIzol (Invitrogen, USA). The RNA was treated with DNase I (Invitrogen, USA) according to the manufacturer's instructions, and then, the total RNA concentration was determined by spectrophotometry at 260 nm. Primers used in this study were designed with Primer 5.0 according to the mouse gene sequence (Table 1). The detailed protocol was referred to as [11]. The amplification reaction is carried out on an ABI Prism 7900HT Sequence Detection System. The expression of relative genes was confirmed with the percentage of the target gene on the control gene by the formula: 2^−(ΔΔCt)^ and Ct = (Cttarget − Ct*β*-actin) treatment − (Cttarget − Ct*β*-actin) control. The relative expression levels of the genes were normalized and expressed as a percentage.

## 3. Data Analysis

All data were shown with the mean ± mean standard error (SEM). The data were performed using SPSS 22.0 software. Differences between means are evaluated using one-way analysis of variance and then Tukey multiple comparisons test if applicable. A *P* value < 0.05 was regarded differences.

## 4. Results

**Figure 1:**
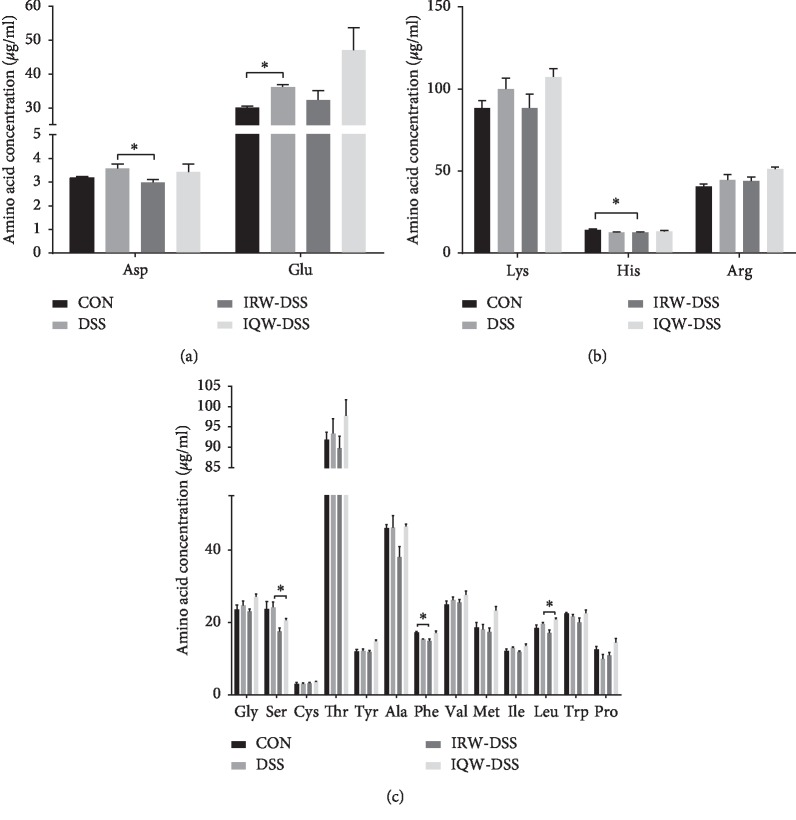
Effect of IRW and IQW on the serum amino acids (*μ*g/mL): (a) acidic amino acids, (b) basic amino acids, and (c) neutral amino acid. Data are given as mean ± SD (*n* = 8). ^*∗*^*P* < 0.05.

Expectedly, DSS injured the mucosal intestinal barrier and resulted in intestinal inflammation. It can increase the expression of inflammatory cytokines in tissues and inhibition of tight junction protein expression. To analyze amino acid metabolomics in blood serum, various amino acids were measured in this study, and the results are shown in Figure 1. The level of Glu improved significantly in the DSS group (*P* < 0.05) relative to the control (Figure 1(a)). Meanwhile, for the IRW-DSS mice, Asp decreased substantially (*P* < 0.05) compared with the DSS mice. On the other hand, for basic amino acids (Figure 1(b)), the IRW-DSS mice have a significant decrease in the level of His (*P* < 0.05) in comparison with the control. Moreover, the neutral amino acid of the IQW-DSS mice improved significantly the level of Leu, but lowered the level of Ser (*P* < 0.05) in relation to the DSS mice (Figure 1(c)). For IRW-DSS mice, Phe decreased substantially (*P* < 0.05) compared with the control mice. No significant differences were observed in the levels of the other amino acids.

**Figure 2:**
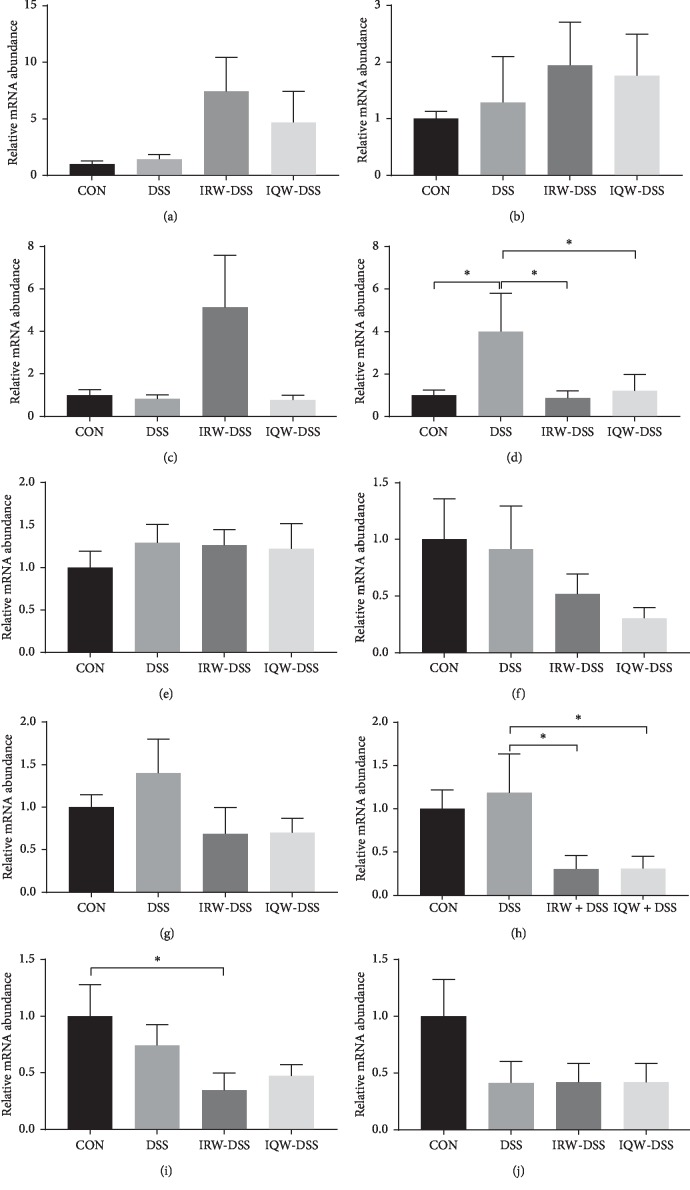
Impact of IRW and IQW treatment on the expressions of colonic inflammatory cytokines. The relative gene expression level determined by RT-PCR of (a) IL-1*β*, (b) IL-4, (c) IL-10, (d) IL-17, (e) IL-18, (f) IL-22, (g) IL-23, (h) TLR4, (i) TNF-*α*, and (j) IFN-g. Data are given as mean ± SD (*n* = 8). ^*∗*^*P* < 0.05.

Contrary to the control group, the level of IL-17 (*P* < 0.05) was reduced in the control mice, IQW-DSS mice, and IRW-DSS mice (Figure 2). The level of TNF-*α* significantly (*P* < 0.05) decreased in IRW-DSS and IQW-DSS mice compared to the DSS mice (Figure 2). Besides, the level of TLR4 (Toll-like receptor 4) reduced significantly (*P* < 0.05) in the IRW-DSS group compared to controls (Figure 2). No other significant differences were observed.

**Figure 3:**
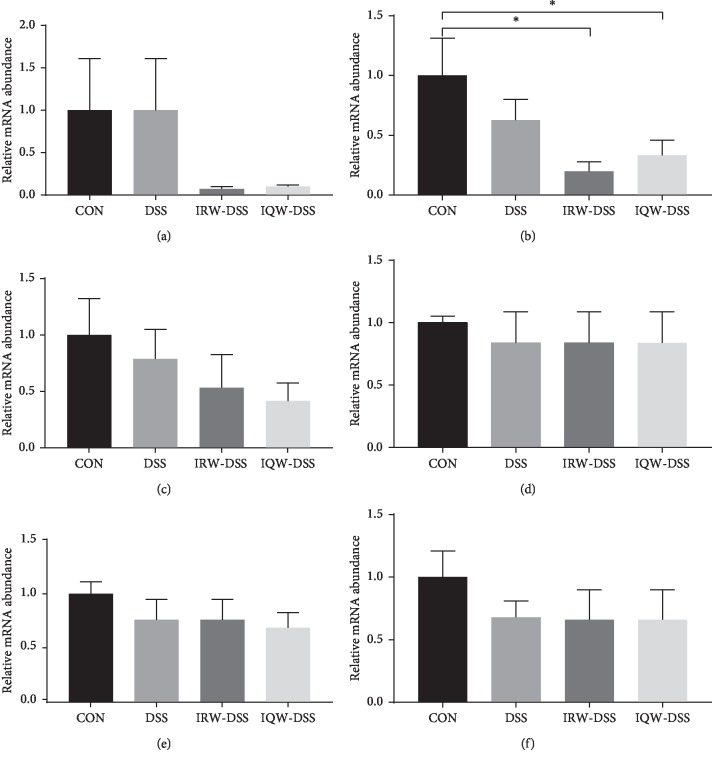
Impact of IRW and IQW treatment on the expressions of barrier function genes. The relative gene expression level determined by RT-PCR of (a) Ocln1, (b) Petp1, (c) Cldn1, (d) Tjp1, (e) Tjp1 m1, and (f) MYD88.

For barrier function genes, compared to control mice, the level of PepT1 (peptide transporter 1) for IQW-DSS mice and IRW-DSS mice decreased substantially (Figure 3). No other significant differences in the level of Ocln1 (occludin-1), Cldn1, Tjp1 (tight junction protein 1), Tjp1 m1, and MYD88 (myeloiddifferentiationfactor-88) were observed.

## 5. Discussion

Inflammatory bowel disease (IBD) is a series of inflammatory responses that occur in the colon and small intestine [19]. IBD may cause diarrhea, abdominal pain, and even gastrointestinal bleeding; when patients suffer from it, this will be a difficult situation to solve [20]. In this study, a model of mouse intestinal inflammation induced by 5% DSS was used to investigate the alleviating effects of IQW and IRW. The results of this study showed that IRW and IQW had an effect on the serum amino acid and reduced the level of IL-17, TNF-*α*, and PepT1.

Amino acids are one of the necessary conditions for the growth of the intestinal tract and the maintenance of mucosal integrity and barrier function [21]. Amino acids can be utilized by intestinal microbes to produce various metabolic products, including proteins, which play a key role in the nutritional and physiological functions of the host [22]. Amino acids are capable of reducing inflammation, oxidative stress, and proinflammatory cytokines [23]. For the DSS mice, the levels of Asp were observed to increase compared with IRW-DSS. Asp possesses the ability of maintaining intestinal tissue integrity and enhancing mucosal energy status by AMPK (adenosine 5′-monophosphate- (AMP-) activated protein kinase) signaling, regulating the expression of proinflammatory cytokine (for instance, TLR4, NODs, and p38) and the apoptosis (for instance, p38 and extracellular signal-regulated kinase 1/2) of intestinal epithelial cells under pathological state [24]. According to our research results, the Glu level of DSS (no treatment) mice was higher than that in the control group (*P* < 0.05). Nevertheless, IQW + DSS mice showed higher Glu levels than the DSS group. Glutamate can reduce the expression of NF-κBp65 and MAPKp38 genes in the intestinal tract, thereby inhibiting the expression of certain pro-inflammatory factors such as TNF-*α*, IL-1*β*, and IL-8 [25]. At the same time, it was found that glutamate could increase the expression of ZO-1, occludin-1, claudin-2, claudin-3, and other tight junction protein, thereby improving the barrier damage caused by intestinal inflammation [26]. In our study, the expression of glutamate in DSS mice increased significantly compared with that in control mice, suggesting that, when the host's gut produces inflammation, more of the glutamate is needed to help relieve symptoms, indicating that it has reduced inflammation and enhanced tissue metabolism. IRW-DSS mice had a substantial decrease in the level of His (*P* < 0.05) in contrast to the control. Some studies have shown that decreased histidine levels can reduce the risk of recurrent ulcerative colitis [27]. In our study, the His levels of IRW mice were decreased, which was a good indication that IRW had a certain therapeutic effect on DSS-induced inflammatory bowel disease.

When using DSS to induce mice to construct an IBD model, cytokines become an important indicator to measure the success of modeling [27]. For the DSS mice, the levels of IL-17 were observed to increase. The most significant effect of IL-17 is its involvement in induction and mediated proinflammatory response [28]. In our study, when DSS mice were treated with IRW and IQW, the expression of IL-17 returned to normal levels. For IBD patients, the expression of proinflammatory cytokines increased significantly. And that includes TNF-*α* and IL-6 [29]. TNF-*α* normally binds to NF-*κ*B and inhibits its translocation, which is then phosphorylated and degraded by IKK, releasing free NF-*κ*B, translocating to the nucleus, and mediating the inflammatory response [30]. In the above results, TNF (tumor necrosis factor) levels in mice treated with IRW and IQW reduced significantly. We seem to find that IRW and IQW treatment can reduce the number of tumors and regulate colitis in mice. These results suggest that these two peptides can inhibit the expression of crude inflammatory cytokines and may be directly involved in the treatment of IBD. On the other hand, the expression of TLR4 and PepT1in IRW mice and IQW mice also supports this view. It is believed that the role of TLR4 signaling in tumors is mainly due to the signaling pathway of proinflammatory cytokines (the expression of which is mediated by TLR) to promote the tumor-promoting effect of microenvironment [31].

As a key mediator of the initiation and progression of CAC, TNF-*α* can significantly increase the number of neutrophils and aggravate the degree of inflammatory infiltration, leading to an increase in the size and number of tumors [32]. There is an inevitable connection between IBD and CAC, and long-term inflammation will inevitably increase the risk of cancer [33]. Abnormal activation of NF-*κ*B is an important cause of CAC progression. Activation of the NF-*κ*B signaling pathway increases cancer cell resistance to therapeutic drugs [34]. IRW and IQW derived from egg protein can inhibit the inflammatory response induced by tumor necrosis factor and the oxidative stress in the endothelial cell [35]. Some studies have found that IRW and IQW have similar effects on IBD, but they are not identical. IRW can inhibit the nuclear translocation of P50 and completely inhibit the NF-*κ*B induced by TNF and thus have anti-inflammation effect. On the other hand, IQW can partially affect the NF-*κ*B signaling pathway by only blocking the translocation of the P50 [13]. And our results show that these two peptides significantly inhibit the expression of TNF-*α*. Therefore, we believe that IRW and IQW can improve serum amino acid content and regulate inflammatory factors to slow the occurrence of inflammation, thereby reducing the risk of CAC development.

## 6. Conclusion

In conclusion, supplemental IRW and IQW reduced the colonic inflammation and barrier impairment in DSS-administered mice. Furthermore, IRW and IQW changed the serum amino acid levels. These results indicated that IRW and IQW are an effective and potentially complementary treatment for IBD. Despite this, further studies are needed to confirm and more profoundly clarify the relationship between IRW and IQW and the gastrointestinal tract along with other parts of the host.

## Figures and Tables

**Table 1:** The primers for this study.

Primers	Product length (pb)	ID	Nucleotide sequence of primers (5′–3′)
Barrier function genes			
Cldn1	107	NM_001244539.1	F: GCCACAGCAAGGTATGGTAAC
			R: AGTAGGGCACCTCCCAGAAG
MYD88	108	NM_010851.2	F: GCATGGTGGTGGTTGTTTCTG
			R: GAATCAGTCGCTTCTGTTGG
Ocln1	208	NM_008756	F: CCTACTCCTCCAATGGCAAA
			R: CTCTTGCCCTTTCCTGCTTT
PepT1	99	NM_214347.1	F: CAGACTTCGACCACAACGGA
			R: TTATCCCGCCAGTACCCAGA
Tjp1 m	64	XM_021187463.1	F: ACCATCATTGTCGTCGCATGTAGATCC
			R: GATGCTCTAGGTGCCTGTTCGTAACG

Cytokine genes			
IL-1*β*	87	NM_001005149	F: GAGCTGAAGGCTCTCCACCTC
			R: ATCGCTGTCATCTCCTTGCAC
IL-4	382	NM_021283.2	F: GAGCCATATCCACGGATGCGACAA
			R: CATGGTGGCTCAGTACTACGAGTA
IL-10	71	XM_021175612.1	F: GCCACATGCTCCTAGAGCTG
			R: CAGCTGGTCCTTTGTTTGAAA
IL-17	119	NM_010552.3	F: TACCTCAACCGTTCCACGTC
			R: TTTCCCTCCGCATTGACAC
IL-18	203	XM_006510028.3	F: AGACAACTTTGGCCGACTTC
			R: CCTTCACAGAGAGGGTCACA
IL-22	73	XM_006513865.3	F: GCTCAGCTCCTGTCACATCA
			R: CACTGTCTCCTTCAGCCTTCT
IL-23	194	NM_031252.2	F: AGTGTGAAGATGGTTGTGAC
			R: CTGGAGGAGTTGGCTGAG
TLR4	64	XM_006509283.3	F: TTCAGAACTTCAGTGGCTGGATT
			R: CCATGCCTTGTCTTCAATTGTTT
TNF-*α*	192	XM_021218154.1	F: AGGCACTCCCCCAAAAGAT
			R: TGAGGGTCTGGGCCATAGAA
IFN-g	361	NM_008337.4	F: ATGAACGCTACACACTGCATCTTGGCTT
			R: CCTCAAACTTGGCAATACTCATGAATGC
*β*-Actin	79	NM_007393.5	F: AACGAGCGGTTCCGATGC
			R: GTAGTTCATGGATGCCACAGG

## Data Availability

All the data are available from the corresponding author upon request.

## Abbreviations

IBD: Inﬂammatory bowel disease
CD: Crohn's disease
UC: Ulcerative colitis
DSS: Dextran sodium sulfate
Trp: Tryptophan
Glu: Glutamic acid
Gln: Glutamine
Ser: Serine
Leu: Leucine
IL: Interleukin
TNF: Tumor necrosis factor
TLR: Toll-like receptor
Cldn1: Claudin-1
MYD88: Myeloid differentiation factor-88
Ocln1: Occludin-1
PepT1: Peptide transporter 1
Tjp1: Tight junction protein 1
AMPK: Adenosine 5′-monophosphate
AMP: Activated protein kinase
NF-*κ*B: Nuclear factor kappa-light-chain-enhancer of activated B cells.
